# Synthesizing implications of a collaborative project on socially sustainable housing policies for the aging population

**DOI:** 10.1186/s13104-023-06565-y

**Published:** 2023-10-19

**Authors:** Susanne Iwarsson, Marianne Granbom, Oskar Jonsson

**Affiliations:** Department of Health Sciences, Lund University, P.O. Box 157, 221 00 Lund, Sweden

**Keywords:** Older adults, Housing in later life, Marginalized groups, Citizen science, Poverty, Disadvantaged neighborhoods, Critical incidents, Housing accessibility

## Abstract

**Objective:**

Based on findings from four transdisciplinary original research studies on housing issues for the aging population, whereof three had a particular focus on marginalized groups, we report a co-produced synthesis of implications from a collaborative research project on socially sustainable housing policies. Researchers and non-academic partners in the ongoing Thematic Collaboration Initiative *Social Rights and Housing for the Aging Population* at Lund University collaborated in co-creative activities aiming at policy recommendations.

**Results:**

Seven types of implications (i.e., themes) that represent macro and meso level approaches with potential to generate impact on social rights and housing for the aging population were identified. The content of legislation and regulations, financial institution strategies, and housing and neighbourhood development exemplify macro level implications. The three themes education and training, communicating with a diversity of stakeholders, and communicating with the public all relate to an overall need for integrated knowledge translation. The theme involving older adults as a resource delivered insight into underutilized capacities of the diverse target group. As the quest for integrated knowledge translation is growing stronger, this research note contributes to development of co-production approaches to synthesize implications of transdisciplinary collaboration, connecting research, practice and policy on societal challenges that ay population aging.

## Introduction

The objective of this research note is to report a co-produced synthesis of implications from a collaborative project on socially sustainable housing policies for the aging population. The synthesis was guided by the question: What changes or refinements of current policies and practices do the synthesised implications suggest?

**Fig. 1:**
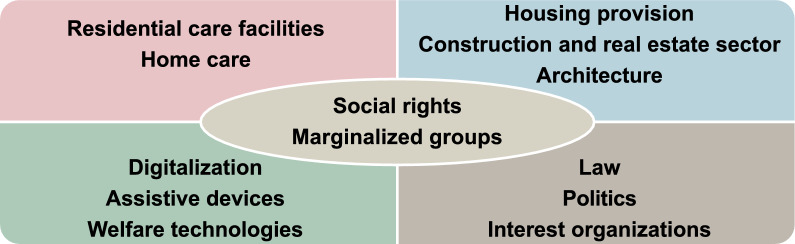
The societal challenge targeted by the Thematic Collaboration Initiative *Social Rights and Housing for the Aging Population*

The overarching aim of the collaborative project is to nurture deepened knowledge exchange and generate new knowledge on housing for the aging population, with a particular focus on marginalized groups, thereby contributing to research-based and socially sustainable housing policies. The research project *Socially Sustainable Housing Policies for People Aging with Disability: Producing a Knowledge Base Supporting Participation and Active Citizenship* was co-developed in the ongoing Thematic Collaboration Initiative (TCI) *Social Rights and Housing for the Aging Population* at Lund University, Sweden (Fig. 1)*.* Our TCI is a hub for knowledge exchange, engaging researchers from four faculties at Lund University and non-academic partners representing public authorities, public and private companies in the housing sector, software developers, and non-profit organizations in joint activities. Subsequently, the project *Older Adults Living in Disadvantaged Areas* [1] was launched, including one of the studies used as part of the basis for this paper.

## Main text

### Four studies elucidating inequalities

Based on joint problem definition involving researchers and non-academic partners in our TCI, built on a mixed-methods convergent design the basis for this paper included four original empirical studies elucidating different perspectives on potential inequalities in the context of housing. Three of the studies had a particular focus on marginalized groups. That is, in two qualitative studies we interviewed women with a low pension and older adults living in deprived neighborhoods, and in the third we explored critical situations involving older tenants in a municipal housing company. The fourth study was a Citizen Science (CS) project with the general population of older adults as the main target group.

#### Women aging with a low pension

Financial security influences good quality housing, health, and longevity, but few recent studies of the financial aspects of aging in place have been published. We explored strategies used to aging in place and thoughts about future housing among women aged 65 and older living with a low pension in Sweden [2]. We conducted semi-structured qualitative interviews with 13 women aged 65 and older with a low pension, followed by thematic analysis. Three themes were generated. "Adjusting to a low pension" explains that most participants needed to supplement their pension with savings or earnings to afford housing and living costs. "The home as a home – and an asset" explains that relocating or renting out the property were common strategies that could raise money to supplement their pension. "Thoughts about the future (home)" explains that most participants preferred to age in place, but pragmatic considerations about potential housing costs, housing type/form, tenure, housing locality, safety, and security were expressed. In addition to the need for further research on this topic, these findings have implications for community planning and can be used to inform pension and housing policies in Sweden and other countries with similar welfare systems.

#### Being older in deprived neighborhoods

The existence of social problems, crime, and a diminishing sense of community are challenges to residents of deprived neighbourhoods. This study explored how older adults in such neighbourhoods in Sweden experience crime and disorder, and how they adapt and respond to these problems and the neighbourhood’s poor reputation [3]. We conducted semi-structured interviews with 22 older adults who had lived 5 years or more in deprived areas, followed by thematic analysis. Most residents were positive about their homes and neighbourhoods, even if criminal acts were part of everyday life. They attempted to manage these events with various strategies. Exit strategies included relocation and forms of adaptation and detachment. They used voice strategies to actively try to solve problems and engage with the community. Loyalty strategies and relativizing were used to defend neighbourhood reputation. The findings show that older adults are not passive victims of their environment; some are active agents in fostering community in deprived neighbourhoods. City improvement programs should extend support to older adults who wish to engage with their neighbourhoods.

#### Critical incidents in municipal housing

Neighbourhood support can improve aging in place for older adults, but research on the role of public housing staff in supporting older tenants is lacking. Twenty-nine participants (janitors and maintenance staff) collected data about critical situations among older tenants residing in municipal multi-family housing. Modifying the Critical Incidents (CI) technique [4] and applying a mixed-methods design, quantitative and qualitative data were collected and analyzed with descriptive statistics and thematic analysis, integrated through narrative [5]. We found that older tenants asked staff for help with daily tasks. The staff identified CI management dilemmas in meeting older tenants' support needs while following the housing company's regulations, maintaining professional responsibilities, respecting individual work attitudes and preferences, and experienced a lack of competencies in some situations. Staff members were responsive to offering support in simple, practical, and emotional situations and in addressing matters they perceived as deficits in social and health services.

#### The housing experiment

To promote active aging and avoid involuntary and unnecessary moves to residential care facilities there is a need for increased public knowledge and raised awareness about accessible housing among older adults. Valid information on environmental barriers in the housing stock is one prerequisite for forward-looking housing provision for the aging population, but CS projects aimed at improving the living conditions of older adults remain rare. For two autumn months in 2021, recruiting in collaboration with the three largest senior citizens associations in Sweden, older adults were invited to contribute to the Housing Experiment (HX) using a smartphone application (app) to report environmental barriers in their dwellings. In all, 1,203 people delivered data to an online open access database. Recruiting a sub-sample to increase the knowledge about the prerequisites for participation in CS we investigated the characteristics of older adults involved, and changes in attitudes and mobile digital literacy after completing HX [6]. Data was collected via online questionnaires before and after HX. Based on a lower response rate than anticipated (approx. 2,500 invited, 147 responded), study participants confirming HX involvement (n = 100) were characterized by mobile digital literacy and high functional ability, and a higher education level than the corresponding general population. A negligible number of them had visited the website displaying the online database. The only significant attitudinal change showed that more rated the importance of housing accessibility lower after involvement in HX compared to those rating it higher. This should be considered in the light of predominantly positive attitudes prior to participating in HX. Further research is warranted to investigate how CS projects could be designed to attract older adults and create conditions for greater gains for the target group.

## Methods

During a 3-year period, experiences and emerging findings from the four studies were presented and discussed at workshops involving researchers and non-academic partner representatives engaged in our TCI in a co-production process. For an example of questions used for small-group discussions in one of the workshops involving researchers (n = 6) and non-academic partner representatives (n = 11), see Table 1. Comments, reflections and suggestions were documented from all workshops arranged.

**Table 1:** Instruction and questions to TCI members

Instruction: Start from the study that is on the table. Discuss based on the questions Write down answers and thoughts in bullet point format on post-it notes
Which parts of the results could be used in society?
Can you see the organization you represent benefiting from the results? How and why? If not, why?
Who else could benefit from the results? - The public, municipal level, national level, organisations, companies? - Practical use—use on the policy level? - Local, regional, national, international? - As knowledge for different categories of staff, including students (= staff of the future)?
In what way could the knowledge be conveyed in a meaningful way to different stakeholders?

Towards the end of the 3-year period, such activities were explicitly aiming at synthesizing the implications of the research initiated by our TCI to nurture the development of socially sustainable housing policies for the aging population.

### Synthesis of implications

The synthesis based on the four studies generated seven types of implications (i.e., themes): legislation and regulations; financial institution strategies; housing and neighbourhood development; involving older adults as a resource; education and training; communicating with a diversity of stakeholders; communicating with the public (for details, see Table 2).

**Table 2:** Synthesis of implications based on four empirical studies

Type of implication	Low pension	Deprived neighbourhoods	Critical incidents	Housing experiment
Legislation and regulations	- Provide grants to incentivize moves from one-family housing to apartments - Change of the proportions pension—housing allowance	- Regulate small housing companies similarly to existing regulation of public housing companies	- Change work environment legislation and related insurances - Attention to good men and their tasks to prevent conflicts with family members	- Inventory of environmental barriers useful to enforce housing companies’ certification of accessibility - Adhere to accessibility requirements for residential care facilities
Financial institution strategies	- Provide mortgages, despite low income - Inform older adults about “smart strategies” regarding amortizing			- Information about whether a dwelling is accessible or not could be useful for banks when providing loans
Housing and neighbourhood development	- Public transport development - Scale up good examples - Develop social meeting places, potentially compensating for needs of larger dwellings - Build more cheaper dwellings - Adapt existing housing rather than building new	- Collect information about residents’ attitudes and experiences to inform architectural planning - Use findings to inform planning, in all kinds of neighbourhoods - Develop strategies for neighbourhood development that do not lead to higher rents - Develop novel housing and tenure alternatives - Strive for neighbourhood integration, counteracting black painting of areas	- Provide digital services included in rents	- Develop housing that responds to demographic change - Make use of data on accessibility to inform construction, retrofits and refurbishment
Education and training			- Housing company staff, landlords and property managers need knowledge and skills to support older tenants and act as a bridge to public authorities	- Data on accessibility can be used for education targeting actors in the housing sector
Communicating with a diversity of stakeholders	- Senior citizens’ organisations can make use of findings for lobbying - Provide politicians with pictures and stories illustrating real-world contexts - Engage with politicians to elicit new ideas, e.g., about sharing economy, collective solutions - Realise older adults as a resource	- Senior citizens’ organisations can make use of findings to exert pressure on municipalities	- Make use of housing company staff as a bridge to municipal support for older adults - Develop back-up function for staff and tenants in housing companies - Highlight and act on the responsibility gap between authorities, companies, and individuals - Incentivize tech companies to provide integrated digital solutions	- Make the app even shorter, to attract municipalities, insurance companies, brokers and builders to inventory accessibility - Approach stakeholders to make advertisements within the app, to support financing it - Involve additional interest organisations
Communicating with the public	- Economic planning for later life is a necessity - Information campaigns about economy strategies	- Elicit media attention based on stories about real-world contexts		- Release the app for public use to support housing decisions
Involving older adults as a resource	- Design public efforts to make it possible to providing rather than receiving support - Introduce rent discounts for tenants providing support to neighbours	- Involve older adults in school and integration projects - Utilize municipal senior citizens’ councils more, for prevention		- Elicit public interest for accessible housing in the prospect of old age

All four studies contributed to implications regarding legislation and regulations, housing and neighbourhood development, and communicating with a diversity of stakeholders. The HX study contributed with findings informing all seven types of implications, and the Low Pension study contributed to all but the implications regarding education and training.

## Discussion

In research as well as in policymaking and practice, the silo-thinking regarding the complex matter of housing for the aging population is striking [7]. That is, on the one hand efforts are concentrated to housing provision, construction and management, and on the other to health care and social services issues—with insufficient communication and coordination between the “hard” and “soft” sectors. During the latest decades, the focus of research on aging has to a considerable extent shifted from diffusion of knowledge to more active processes of utilization of research results [8]. The collaborative project underlying this research note is novel in several respects. Applying an innovative combination of research methods, collaboration endeavours and strategies for synthetization of implications, our TCI and the collaborative project contribute to closing the gap between research, policy and practice, in different sectors of society. Our project is transdisciplinary in nature, implying a fusion of academic interdisciplinary knowledge [9] with the knowledge of professional practitioners and lay people [10], involving them in co-production of a synthesis of implications from four original studies. Thus, our approach to collaboration responds to the need for bottom-up transdisciplinary approaches in which older adults and non-academic partners are involved [11, 12], where a diversity of knowledge and service users interact with researchers from different disciplines in transdisciplinary co-creation of knowledge.

The synthesized implications generated from four transdisciplinary empirical studies of different character is a sound foundation towards a knowledge base supporting participation and active citizenship in the diverse aging population. Based on collaborative activities involving researchers and non-academic partner representatives, Table 2 highlights seven types of implications that represent macro and meso level approaches with potential to generate impact on social rights and housing. Exemplifying macro level implications [10], the three themes legislation and regulations, financial institution strategies, and housing and neighbourhood development had such content. The three themes education and training, communicating with a diversity of stakeholders, and communicating with the public all relate to an overall need for co-creation [13] and integrated knowledge translation [14]. Finally, the theme and strategy involving older adults as a resource delivered insight into underutilized capacities of the diverse target group, as described by James and Buffel [15].

Given the ambition to address social rights in the context of housing for the aging population, the four original empirical studies used as a basis for this paper did deliver important insights regarding specific prerequisites related to marginalized groups and socio-economic diversity. For example, Taei et al.’s study [3] elucidated that older adults living in deprived neighborhoods are not necessarily vulnerable because vulnerability is more related to sense of exclusion or not feeling part of a community. Rather, older adults who feel included and committed to the community make efforts to contribute to improving their neighborhoods. Turning to Frögren et al.’s study [6], attempting to understand requirements to motivate older adults to participate in CS about matters that likely concerns them, the findings show that it is challenging to reach those with lower education and less digital literacy. Still, it should be noted that such aspects did not end up as prominent in the synthesized implications (see Table 2). This could likely be attributed to insufficient diversity in the TCI partnership, which requires specific attention in future endeavors of this kind.

In conclusion, as the quest for integrated knowledge translation is growing stronger, researchers need to develop strategies for true collaboration involving non-academic partners. This research note contributes to the development of co-production approaches to synthesize implications of transdisciplinary collaboration, connecting research, practice and policy contexts where a diversity of actors is struggling to meet the societal challenges that accompany population aging.

## Limitations

As the authors of this research note all are researchers, no non-academic partner representative has co-authored this paper. Even if none of those fulfilled the criteria required for scientific authorship, the content was strongly influenced by their input throughout our TCI activities. For transparency, a draft version of the manuscript was shared with partner representatives in our TCI Steering Group prior to submission, encouraging them to comment on the content.

## Data Availability

Data collected for this research note will, after de-identification, be available on reasonable request. The prerequisite for this is a data transfer agreement, approved by legal departments of the institutions of both the requesting researcher and the researchers that provided data for the study. Proposals should be directed to: susanne.iwarsson@med.lu.se.

## Abbreviations

CI: Critical incidents
CS: Citizen science
HX: Housing experiment
TCI: Thematic collaboration initiative
